# *In Vivo* Imaging Reveals Distinct Inflammatory Activity of CNS Microglia versus PNS Macrophages in a Mouse Model for ALS

**DOI:** 10.1371/journal.pone.0017910

**Published:** 2011-03-18

**Authors:** Payam Dibaj, Heinz Steffens, Jana Zschüntzsch, Fabien Nadrigny, Eike D. Schomburg, Frank Kirchhoff, Clemens Neusch

**Affiliations:** 1 Department of Neurogenetics, Max-Planck-Institute for Experimental Medicine, Göttingen, Germany; 2 Institute of Physiology, University of Göttingen, Göttingen, Germany; 3 Department of Neurology, University of Göttingen, Göttingen, Germany; 4 U862, Institut François Magendie, Bordeaux, France; 5 Department of Molecular Physiology, University of Saarland, Homburg, Germany; 6 Department of Neurology, University of Ulm, Ulm, Germany; National Institute on Aging Intramural Research Program, United States of America

## Abstract

Mutations in the enzyme superoxide dismutase-1 (SOD1) cause hereditary variants of the fatal motor neuronal disease Amyotrophic lateral sclerosis (ALS). Pathophysiology of the disease is non-cell-autonomous: neurotoxicity is derived not only from mutant motor neurons but also from mutant neighbouring non-neuronal cells. *In vivo* imaging by two-photon laser-scanning microscopy was used to compare the role of microglia/macrophage-related neuroinflammation in the CNS and PNS using ALS-linked transgenic SOD1^G93A^ mice. These mice contained labeled projection neurons and labeled microglia/macrophages. In the affected lateral spinal cord (in contrast to non-affected dorsal columns), different phases of microglia-mediated inflammation were observed: highly reactive microglial cells in preclinical stages (in 60-day-old mice the reaction to axonal transection was ∼180% of control) and morphologically transformed microglia that have lost their function of tissue surveillance and injury-directed response in clinical stages (reaction to axonal transection was lower than 50% of control). Furthermore, unlike CNS microglia, macrophages of the PNS lack any substantial morphological reaction while preclinical degeneration of peripheral motor axons and neuromuscular junctions was observed. We present *in vivo* evidence for a different inflammatory activity of microglia and macrophages: an aberrant neuroinflammatory response of microglia in the CNS and an apparently mainly neurodegenerative process in the PNS.

## Introduction

Amyotrophic lateral sclerosis (ALS) is an adult-onset neurological disorder characterized by progressive loss of upper and lower motor neurons and degeneration of pyramidal tracts. Transgenic mice expressing various human ALS-linked mutations in the gene encoding the enzyme superoxide dismutase-1 (mSOD1) mimic to some extent the fatal paralysis seen in patients [1], [2], [3], [4], [5], [6]. Dominant mutations in SOD1 are, so far known, the most frequent cause of familial ALS (fALS). In addition, the best-studied animal model of fALS is that caused by mutations in SOD1 [7]. Also in sporadic ALS (sALS), the analysis of the structure of amyloid fibrils [8] and the presence of misfolded SOD1 in the spinal cord of sALS patients [9] suggest an involvement of SOD1 in the pathophysiology of the disease. In addition, recent clinical and electrophysiological data show that the human SOD1-G93A phenotype closely resembles sALS suggesting comparable disease pathology [10].

The mSOD1-mediated toxicity is non-cell-autonomous deriving not only from motor neurons but also from neighbouring glia. In particular, microglia and astrocytes substantially contribute to motor neuron death and disease progression [4], [5], [6], [11], [12], [13], [14], [15]. In the case of microglia, selective silencing of the mutant gene in the innate immune cells of the CNS and in macrophages in a SOD1-model has substantially slowed disease progression [4], [5]. In addition, replacement of mSOD1-expressing cells of the myeloid lineage including microglia by non-mutated cells via bone marrow transplantation equally slowed disease progression [11]. These studies strongly emphasize a microglial contribution to the propagation of motor neuron death. Despite this evidence the impact of neuroinflammation to disease progression is still a matter of debate and little is known about the functional state of mSOD1-expressing microglia during disease progression.

In addition, recent evidence suggests that loss of peripheral axons and neuromuscular synapses is a primary event in motor neuron degeneration, even before the respective lower motor neuron cell bodies show signs of degeneration [16], [17]. The impact of neuroinflammation on axonal degeneration in the peripheral nervous system is, however, still elusive.

Microglia as the monocyte-lineage immune effector cells of the CNS play central roles in CNS neuroinflammation. In the resting state, microglia display highly dynamic process movements to constantly survey their microenvironment in brain [18], [19] and spinal cord [20], [21], [22]. This spontaneous activity enables microglia to rapidly respond to injuries within nervous tissue [18], [19], [20], [21]. Following acute tissue injury, surrounding microglia extend processes towards the injured site within minutes while retracting opposite processes: a phenomenon called polarization. Within a few hours following subsequent ameboid transformation, responding microglia begin migrating and commence phagocytosis. This injury-induced transformation of microglia to reactive states is known as microglial activation [23].

Here, we used time-lapse 2-photon laser-scanning microscopy (2P-LSM) and transgenic mouse technology to investigate microglia and macrophage reaction during disease course in the SOD1-G93A (SOD1^G93A^) mouse model for ALS. The use of transgenic mice containing both fluorescent microglia/macrophages [24] and neurons [25] enables us to correlate mutation-induced changes of microglia/macrophage function with the progressive axonal degeneration in the CNS or PNS, respectively. Since the stages of microglial activation are in part defined by their response to injury, including polarization, migration and phagocytosis [23], we also investigated the reaction of microglia to axonal transection in different stages of SOD1^G93A^ mice. In a second approach, the microglia-related macrophages of the PNS were investigated using the same paradigms. The *in vivo* imaging approach allows us to combine morphological changes with cellular behavior in an intact environment under baseline conditions as well as in response to focal lesions. Since the maintenance and local expansion of mSOD1-microglia is dependent on self-renewal within the CNS, we refer to microglia as the resident monocyte-lineage cells of the CNS [26].

CNS microglia showed substantial inflammatory activity. In preclinical stages, microglial cells that highly reacted towards focal lesions acquired over the course of the disease an ameboid shape indicating morphological transformation and consequent cellular activation. In clinical stages activated microglia lost their injury-directed response. In contrast to microglia-mediated neuroinflammation in the CNS, morphologically pro-inflammatory reactions of macrophages were negligible in the PNS suggesting primarily passive roles of macrophages in axonal degeneration.

## Materials and Methods

### Ethics statement

The experiments were performed according to the ethical guidelines of the national animal protection law and were authorized by the ethical committee of the State of Lower Saxony (review board institution: Niedersächsisches Landesamt für Verbraucherschutz und Lebensmittelsicherheit, Dezernat 33, Oldenburg, Germany; approval-ID: 509.42502/01-39.03).

### Mouse strains

Transgenic TgN(SOD1-G93A)G1H mice (The Jackson Laboratory, Bar Harbor, USA) were maintained as hemizygotes by mating transgenic males with B6SJL hybrid females. Hemizygote male TgN(SOD1-G93A)G1H mice were crossbred with female TgH(CX3CR1-EGFP)xTgN(THY1-EYFP) mice to obtain SOD1^G93A^ mice with fluorescently labeled macrophages/microglia and projection neurons. TgH(CX3CR1-EGFP)xTgN(THY1-EYFP) mice were previously obtained by crossbreeding homozygous CX3CR1-EGFP mice, in which the expression of the green fluorescent protein EGFP in monocytes, macrophages and microglia is achieved by placement of the EGFP reporter gene into the *Cx3cr1* locus encoding the chemokine receptor CX3CR1 [24], with transgenic THY1-EYFP mice expressing the yellow fluorescent protein EYFP in projection neurons and their respective axons [27]. TgH(CX3CR1-EGFP) mice and TgN(THY1-EYFP) mice were of B6SJL background for more than 10 generations. The corresponding littermates (hemizygotes with respect to all three genetic alterations) were used to study microglia-axon interactions *in vivo* during the disease course of SOD1^G93A^ by discerning their cell-type specific fluorescent protein expression simultaneously.

The experiments were carried out on adult mutant mice (preclinical: 60 and 75-day-old; onset of disease: 90-day-old; clinical: 105 and advanced clinical: 120-day-old). Non-transgenic littermates were used as controls. In control mice, no age-related changes in microglial behavior were observed. Even though female SOD1^G93A^ expressing mice survive a few days longer than male mutant mice, gender-dependent differences in microglial morphology and reactions were not observed. Similarly, there were no gender-dependent differences in the behavior of macrophages in the PNS. Mice were kept in the mouse facility of the institute according to national and European guidelines for the welfare of experimental animals.

### Immunohistochemistry

The animals were anesthetized (80 mg pentobarbital sodium per kg body weight i. p.) and perfused transcardially with PBS followed by 4% paraformaldehyde. After tissue processing, 20 µm-thick cryosections were cut from the lumbar spinal cord. After thawing and rehydration, the sections were incubated with blocking buffer (10% normal goat serum and 0.1% Triton X-100) followed by primary antibody (SMI32, 1:1000; purchased from Sternberger Monoclonal Incorporated, Lutherville, USA). Samples were incubated with a fluorescent Cy-3 conjugated secondary antibody (1:500; Jackson Immunoresearch, West Grove, PA, USA). Confocal images were obtained using a Zeiss Axiovert 200M/LSM 510 Meta confocal laser-scanning microscope (Zeiss, Jena, Germany) equipped with argon (488 nm) and HeNe (543 nm) laser.

### Anesthesia and surgery

The experiments were carried out on mice of different clinical stages of SOD1^G93A^ and on control mice of corresponding age (60 to 140 days of age) under general anesthesia initiated by 80 mg pentobarbital sodium i. p. (dissolved in 0.9% NaCl) per kg body weight. After cannulation of the jugular vein, anesthesia was continued with 40-60 mg per kg and h methohexital sodium (Brevimytal, Hikma, London, UK). A tracheotomy was performed and a tracheal tube was inserted for artificial ventilation. Spinal cord segments L4 and L5 were exposed by laminectomy of the spines L1 to L3 for image recording of the dorsal column. Recording of the lateral column was achieved by turning the mice by 80 degrees. To avoid movement caused by active respiration during imaging, the animal was paralyzed with pancuronium bromide (Pancuronium Organon, Essex Pharma GmbH, Munich, Germany; 800 µg per kg and h by i. p. injection every hour) and artificially ventilated with a gas mixture of CO_2_ (2.5%), O_2_ (47.5%), and N_2_ (50%) at 120 strokes/min (100-160 µl/stroke depending on the body weight). In addition, spinal column was rigidly fixed with two custom-made clamps, each having two joints for keeping the mouse in the turned position.

For exposing proximal peripheral nervous tissue including spinal roots and dorsal ganglia, the dura was carefully removed without setting a lesion to the spinal cord. In all experiments, the exposed spinal cord was continuously superfused with ACSF (artificial cerebro-spinal fluid: 125 mM NaCl, 25 mM NaHCO_3_, 2.5 mM KCl, 1.25 KH_2_PO_4_, 1 mM MgCl_2_, 2 mM CaCl_2_*H_2_O and 10 mM glucose). For recordings of peripheral nerves and NMJs of the hind limbs, sciatic nerve and musculus tenuissimus were exposed, respectively. For this purpose, spinal column and the respective foot, each, were fixed with a clamp. For labeling of blood vessels, we injected Texas Red-Dextran (70 kDa, 1.25% w/v, Invitrogen) through a second central venous catheter after cannulation of the other jugular vein. After an initial injection of 50 µl bolus (5 min. before the start of the experiment), application of Texas Red was continued with 50 µl per hour. Rectal body temperature was measured and kept between 36 and 38°C by a heated plate. Electrocardiograms were monitored throughout the experiment. A heart rate below 420 per min was taken as sign for sufficient anesthesia [20], [21]. Furthermore, the dose of methohexital used in pancuronium-paralyzed mice was the same dose we previously used in non-paralyzed mice with corresponding weight. The depth of anesthesia was tested by provoking the corneal reflex and reactions to noxious stimuli.

### 2-Photon laser-scanning microscopy and image acquisition

Photographic and epifluorescence overviews of the spinal cord were acquired using a 5× (NA 0.15) objective (Carl Zeiss GmbH, Jena, Germany) and a CCD camera. High resolution *in vivo* imaging was performed using a commercial two-photon laser-scanning microscope (2P-LSM, Zeiss Axiocope 2 with LSM510 NLO scanhead) equipped with a fs-pulsed, mode-locked titanium-sapphire infrared laser (Mira 900/10 W Verdi; Coherent, Glasgow, UK) or a custom-made 2P-LSM equipped with a fs-pulsed titanium-sapphire laser (Chameleon Ultra II; Coherent). For 2P-recordings, a Zeiss W N-Achroplan 40× (NA 0.75) or a Zeiss W Plan Apochromat 20× (NA 1.0) water immersion objective was used. For excitation, the laser was set at 895±5 nm for the simultaneous excitation of EGFP and EYFP. Emitted light was split by a 520 nm longpass dichroic mirror (Semrock, Rochester, USA) and collected by photo-multiplier tubes (Hamamatsu, Japan) through two bandpass filters: a 494±20.5 nm (FF01-494/41-25) and a 542±25 nm (FF01-542/50-25 (Semrock). The fluorescence of Texas Red was separated from the EGFP or EYFP signal by a 560 nm long pass filter (Carl Zeiss) and a HQ575 emission filter (AHF Analysentechnik, Tübingen, Germany). The relations of the measured fluorescence intensities were comparable between the two microscopes. Parallel, uniformly spaced (1.5 to 2.4 µm) planes of 100*100 to 600*600 µm^2^ regions were recorded, digitized and processed to obtain z-stacks of images (256×256 to 1024×1024 pixels in size). Voxel sizes ranged from 0.2×0.2×1.5 to 1.17×1.17×2.4 µm for the xyz-axes. The total acquisition time for a stack of 15 to 30 images was approximately 1-2 min. Recordings of, at most, 100 µm stack depth were obtained. Several stacks were taken continuously to get time-lapse series of microglial action. Standardized lesions were applied by the titanium-sapphire laser focused for 2 seconds to a plane of an axon diameter until the fluorescence signal began increasing. Only morphologically intact axons were chosen for the experiments.

### Image processing and morphometric analysis

Image processing and morphometric analysis were performed using the Zeiss LSM software, ImageJ (http://rsbweb.nih.gov/ij) and Matlab (version 7, MathWorks, Ismaning, Germany). Statistical analysis was performed using Origin 7 software (Northampton, USA). Prior to any analysis, the time series of image stacks were corrected for the shifts in the horizontal and vertical directions by using an autocorrelation based on custom-made software written in Matlab (v.7). In the majority of images, detector noise was removed by the median filter of ImageJ. The recorded stacks are shown in maximum intensity projections (MIPs). Although EGFP and EYFP signals cannot be separated without spectral unmixing, the filter sets were chosen in a way that permitted an unambiguous distinction of EGFP expressing microglia from EYFP expressing neurons. The cellular differentiation was facilitated by the different morphologies of axons and microglia and the large predominance of EGFP signal in one channel. Decrease and increase of axonal and microglial fluorescence signals, respectively, was measured by area fraction analysis (ImageJ) of particles in comparable regions using the corresponding channels. Prior to that, proper precautions were taken to ensure that respective fluorescence signals were similar in different control mice or in different SOD1^G93A^ mice of the same stage. The number of axons in comparable regions was measured by plot profile analysis (ImageJ). Ramification analysis referred to the main processes directly originating from the microglial cell body [20]. To determine the motility of microglial processes, changes of the length of microglial processes were determined in relation to the control situation before lesion and after laser-induced injury. The outermost processes comprising protrusions at their end were quantified. Measurements of the intracellular area of single macrophages in MIPs were performed using Zeiss LSM software. Care was taken to avoid measurements of multiple cells, e. g. no measurements were performed in macrophage cell clusters. With regard to ramification analysis and to measurements of the intracellular area, at least three exemplary cells were analyzed per mouse. For quantification purposes, we defined increases in fluorescence located directly around the injured site as the microglial response [18], [20]. Increases in fluorescence attributed to process ingrowth or soma migration were not distinguished. We used the function *R*(*t*) = (*R*_x_(*t*) − *R*_x_(0))/*R*_y_(0) as described [18]. For this purpose, we used the Analyze Particle function of ImageJ to measure the microglial density around an injury. Diameters for outer (y) and inner (x) area were 70 µm and 35 µm, respectively. We included only comparable lesions in the analysis, indicated by the average size of the autofluorescence signal. The rate of engulfments was equally measured in comparable regions (MIPs; xy-size about 250x250 µm^2^) in four preclinical, four clinical mice and four controls.

### Statistical analysis

For statistical analysis Origin 7 software (Northampton, USA) was used. Mean values were given ± standard of the mean (SEM). Statistical significance (p<0.05) was determined using ANOVA followed by Tukey test.

## Results

*In vivo* 2P-LSM was applied to simultaneously record central axons of projection neurons and microglia in the spinal cord [20], [21]. In a second approach, lower motor axons and macrophages in spinal roots, peripheral nerves as well as neuromuscular junctions were investigated.

### Axon degeneration and microglia activation in lateral spinal cord of SOD1^G93A^

**Figure 1:**
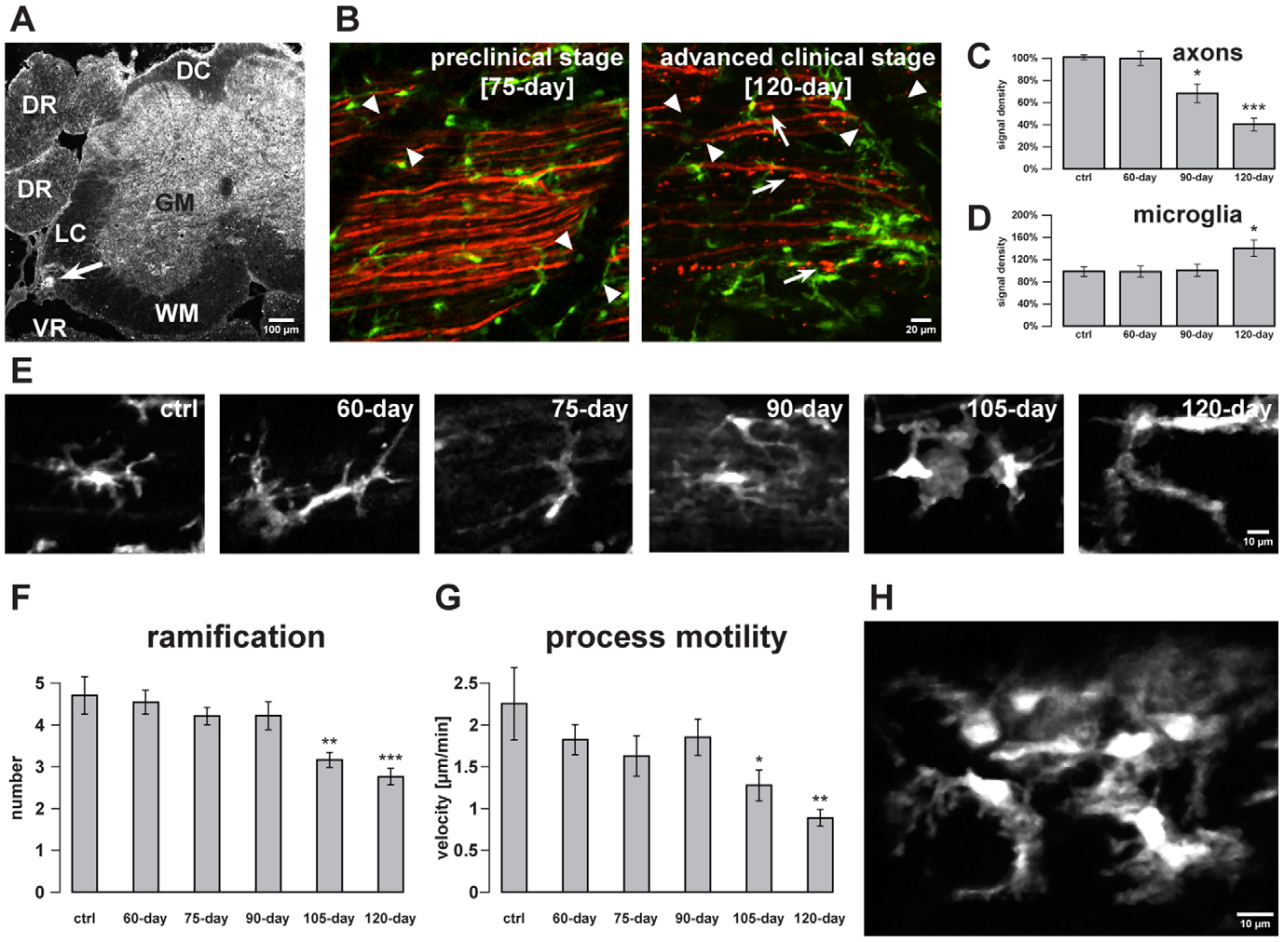
Axonal degeneration and microglial activation in the lateral column of the spinal cord. Spinal cord microglia cells were recorded, shown in part with neighbouring axons, within the lateral column during the disease course of SOD1^G93A^. The experiments were performed in double transgenic mice expressing EGFP in microglia and EYFP in projection neurons. For better visualization, EYFP fluorescence is depicted with a red color table in all images. All two-photon images are arranged such that rostral is to the left side. ***A***, confocal image of a SMI32-stained cross section of spinal cord. Motor neurons were stained in the ventral part of the lumbar spinal cord. The autofluorescence of a lesion (arrow) induced by a two-photon laser pulse before perfusion of the mouse indicates the area of *in vivo* imaging in the lateral column of the spinal cord. ***B***, Time course of axonal degeneration in SOD1^G93A^ mice. Recordings were obtained in preclinical (75-day-old) and advanced clinical (120-day-old) stages in comparable regions of the lateral spinal cord. In 120-day-old mutant mice axons were mostly swollen and interrupted (some exemplary axons are marked by arrows). Note the green fluorescence of monocytes inside the vessels (vessel boundaries marked by arrowheads). ***C***,***D***, Quantification of axonal and microglial signal density, respectively, in comparable regions of the lateral spinal cord during the course of disease from preclinical stage (60-day-old), onset of disease (90-day-old) to advanced clinical stage (120-day-old). ***E***, Continuous change of microglial morphology towards a more ovoid and a less ramified shape from preclinical stages (60-day-old and 75-day-old), onset of disease (90-day-old) to clinical and advanced clinical stages (105-day-old and 120-day-old). ***F***,***G***, Quantification of microglial ramification and process motility during the same stages of disease as shown in ***E***. ***H***, An exemplary microglia cell cluster is depicted in a clinical stage (105-day-old). Values are presented as mean ± SEM; statistical significance determined by using ANOVA followed by Tukey test (*p<0.05, **p<0.01, ***p<0.001). GM, grey matter; WM, white matter; DC, dorsal column; LC, lateral column; DR, dorsal root; VR, ventral root.

Under general anesthesia and after exposure of the spinal cord segments L4 and L5 by laminectomy of the spines L1 to L3, a laser-induced lesion was set to the lateral column of the spinal cord (Fig. 1*A*). Using this approach, we were able to record a part of the spinal cord under *in vivo* conditions which largely carries out efferent, i. e. motor functions.

In rodents, different from the human anatomy, axons of the corticospinal tract (CST) are located in both the deep part of the dorsal column as well as the lateral column of the spinal cord. Affected upper motor axons are predominantly observed in the lateral column of mSOD1 mice [28]. Here, mainly descending axons including approximately 20% of all CST axons are located. Additionally, early vascular changes leading to neurotoxic microhemorrhages, indicated by hemosiderin deposits, exist to a greater extent in the lateral white matter of the spinal cord than in the dorsal or ventral white matter [29]. A high number of degenerated, interrupted and swollen axons were found in the lateral column of the lumbar spinal cord in clinically affected SOD1^G93A^ compared to preclinical animals (Fig. 1*B*). Accordingly, signal density of EYFP-labeled axons in comparable regions was reduced during disease course from preclinical 60-day-old mice (set as 100±6.6%, n = 6 mice) to 90-day-old (onset of disease, 68.9±8.6%, n = 6, p<0.05) and to 120-day-old animals (advanced clinical stage, 41.2±5.9%, n = 6, p<0.001) (Fig. 1*C*). The signal density in 60-day-old mice was comparable to that in control mice (101±2.6%, n = 8) (Fig. 1*C*). The number of axons in comparable regions was also reduced during the disease course from 60-day-old mice (set as 100±11.9%, n = 5 mice) to 90-day-old (44.3±5.5%, n = 4, p<0.01) and to 120-day-old mice (25.9±2%, n = 5, p<0.001). The number of axons in 60-day-old mice was comparable to that in control mice (102.5±7%, n = 6).

Coinciding with axonal degeneration, signal density of EGFP-labeled microglia in the respective areas significantly increased with the age of the mouse from 60-day-old (set as 100±10.8%, n = 6 mice) to 90-day-old (102.5±11.6%, n = 6) to 120-day-old animals (141.7±15.5%, n = 6, p<0.05) (Fig. 1*D*). The signal density in 60-day-old mice was comparable to that in control mice (100.2±9.6%, n = 8; Fig. 1*D*). As shown previously in Iba1- and CD11b-stained lumbar tissue, the number of activated microglia increases during disease progression [4]. Furthermore, a substantial increase in the number of activated microglia is observed in the ventral horn of the lumbar spinal cord in clinical stages indicated by MHCII-staining [30]. In support with these earlier studies, the number of EGFP-labeled microglia in the respective areas significantly increased with the age of transgenic mice from 60-day-old (set as 100±9.4%, n = 5 mice) to 90-day-old (118.3±7.6%, n = 4) and to 120-day-old animals (173.3±24.7%, n = 5, p<0.05). The number of microglia in 60-day-old mice was comparable to that in control mice (97.7±12.5%, n = 6).

During the course of the SOD1^G93A^ disease, microglial cells in the spinal cord lateral column underwent a continuous change of their morphology towards an ameboid-like shape, indicating ongoing inflammatory activity (Fig. 1*E*). The number of microglial processes extending from the cell soma (so-called first order processes) continuously decreased from preclinical stages (60-day-old: 4.55±0.3, n = 15 cells in 5 mice; 75-day-old: 4.22±0.22, n = 12 in 4 mice) and onset of disease (90-day-old: 4.23±0.35, n = 18 in 6 mice) to clinical stages (105-day-old: 3.18±0.19, n = 23 in 6 mice, p<0.01; 120-day-old: 2.78±0.21, n = 32 cells in 8 mice, p<0.001) (Fig. 1*F*). The number of first order processes of preclinical microglia was comparable to that of control microglia (4.71±0.46, n = 16 in 6 mice) (Fig. 1*F*).

In addition, baseline motility of microglial processes slowed by nearly 50% in the same time course (60-day-old mice: 1.83±0.19 µm/min, n = 36 microglial processes in 5 mice; 75-day-old: 1.63±0.25 µm/min, n = 26 in 4 mice; 90-day-old: 1.86±0.23 µm/min, n = 41 in 6 mice; 105-day-old: 1.29±0.19 µm/min, n = 41 in 6 mice, p<0.05; 120-day-old: 0.9±0.11 µm/min, n = 45 in 8 mice, p<0.01) (Fig. 1*G*). Microglial processes of 60-day-old to 90-day-old mice displayed comparable baseline motility to processes of control microglia (2.26±0.44, n = 33 in 6 mice). Process retractions similarly slowed during the disease course (control: −2.25±0.39, n = 33 in 6 mice; 60-day-old mice: −1.88±0.14 µm/min, n = 36 in 5 mice; 75-day-old: −1.71±0.25 µm/min, n = 26 in 4 mice; 90-day-old: −1.82±0.2 µm/min, n = 41 in 6 mice; 105-day-old: −1.33±0.16 µm/min, n = 41 in 6 mice, p<0.05; 120-day-old: −1±0.08 µm/min, n = 45 in 8 mice, p<0.001).

Unlike WT-microglia, which mutually repel each other when neighbouring cells encounter one another [19], mutant microglia formed cell clusters in clinical stages of SOD1^G93A^ (Fig. 1*H*).

### Highly reactive preclinical SOD1^G93A^ microglia lose their target-directed response in clinical stages

**Figure 2:**
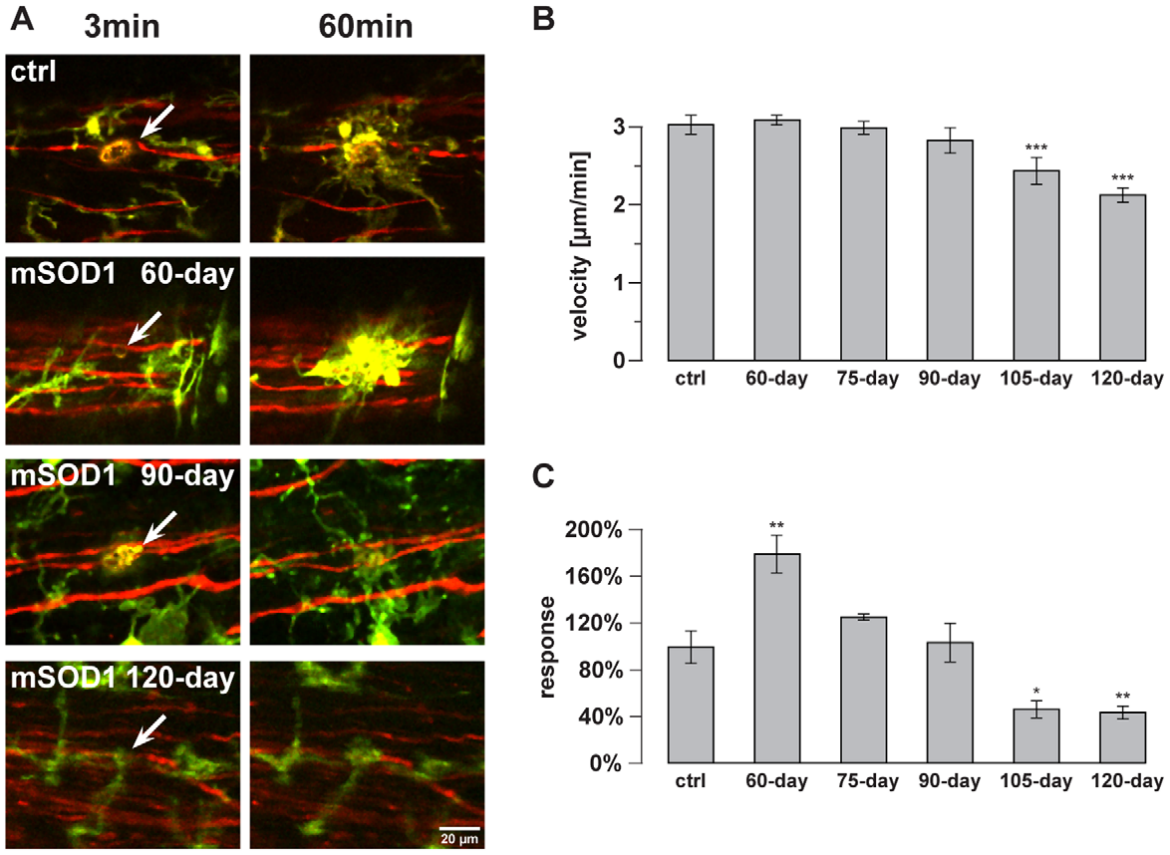
Microglial response in the lateral column of the spinal cord during SOD1^G93A^ disease course. Microglial reaction towards laser-induced single axon transections within the lateral column of the spinal cord during SOD1^G93A^ disease course. ***A***, Left images were taken 3 min after axonal transection (autofluorescence and arrow) in control and mutant [preclinical 60-day-old, 90-day-old (onset of disease) and advanced clinical 120-day-old] mice. Images on the right were taken 60 min post lesion. See also Videos S1, S2, S3, S4, S5. ***B***,***C***, Quantification of the velocity of microglial process movements and of microglial lesion response (increase of EGFP fluorescence in a defined area around the injury) to the injured site from preclinical stages (60-day-old and 75-day-old), onset of disease (90-day-old) to clinical and advanced clinical stages (105-day-old and 120-day-old). Control mice were of corresponding age (60 to 140 days of age). Values are presented as mean ± SEM; ANOVA followed by Tukey test (*p<0.05, **p<0.01, ***p<0.001).

In the next set of experiments, we analyzed the microglial response towards laser-mediated axonal injury during disease course. In Fig. 2*A*, the microglial reaction towards a single axon transection in the spinal lateral column is demonstrated in controls (Videos S1 and S2) compared to SOD1^G93A^ mice in different disease stages (preclinical stage at 60-day-old, onset of disease at 90-day-old and advanced clinical stage at 120-day-old mice; Videos S3, S4, S5). Coinciding with a slowed baseline motility of microglial processes in clinical stages (Fig. 1*G*), the velocity of process extensions towards the lesion site was also significantly decreased in these stages (105-day-old: 2.44±0.18 µm/min, n = 42 microglial processes in 5 mice, p<0.001; 120-day-old: 2.13±0.1 µm/min, n = 61 in 7 mice, p<0.001) compared to controls (3.03±0.13 µm/min, n = 63 in 7 mice) (Fig. 2*B*). The latter was comparable with lesion-induced process movements in preclinical stages and at disease onset of SOD1^G93A^ (60-day-old: 3.09±0.07 µm/min, n = 46 in 5 mice; 75-day-old: 2.99±0.09 µm/min, n = 35 in 4 mice; 90-day-old: 2.83±0.17 µm/min, n = 36 in 4 mice) (Fig. 2*B*).

Furthermore, single axon transections led to a reduced response (increase in fluorescence located directly around the injured site) of microglia towards the lesion site in clinical stages (105-day-old: 47.2±7.8%, n = 5 mice, p<0.05; 120-day-old: 44.4±6.1%, n = 6, p<0.01) compared to control mice (set as 100±14.2%, n = 7) (Fig. 2*C*; Videos S1, S2 and S5). At disease onset (90-day-old: 103.8±16.9%, n = 4), microglial response was similar to controls (Fig. 2*C*). Coinciding with ameboid transformation in clinically affected mice, SOD1^G93A^ microglia partly lose their directed response towards lesioned tissue. Ameboid microglia, however, often displayed permanent spontaneous activity with phagocytosis of morphologically unaffected tissue (Video S6). This included permanent ameboid migration and phagocytic activity. The rate of spontaneous engulfments by microglial processes (13.3±3.9 engulfments in 250 µm×250 µm in 30 minutes, n = 4 mice, p<0.05) was significantly higher than in control mice (1.75±0.25, n = 4) or preclinical SOD1^G93A^ mice (2±0.6, n = 4).

Microglial reaction towards the lesion site, however, was distinctly increased in preclinical mice (60-day-old: 179.1±16.6%, n = 5 mice, p<0.01; 75-day-old: 125.5±3.2%, n = 4; compared to controls, set as 100±14.2%, n = 7) (Fig. 2*C*; Videos S1, S2, S3, S4). This increased response of microglia comprised a rapid movement of cell somata towards the lesion site (in some cases even within a few minutes after the lesion) combined with early phagocytic activity (Fig. 2*A*; Videos S3 and S4). The response of preclinical microglia included engulfments within and around the site of the laser-induced axonal transection.

**Figure 3:**
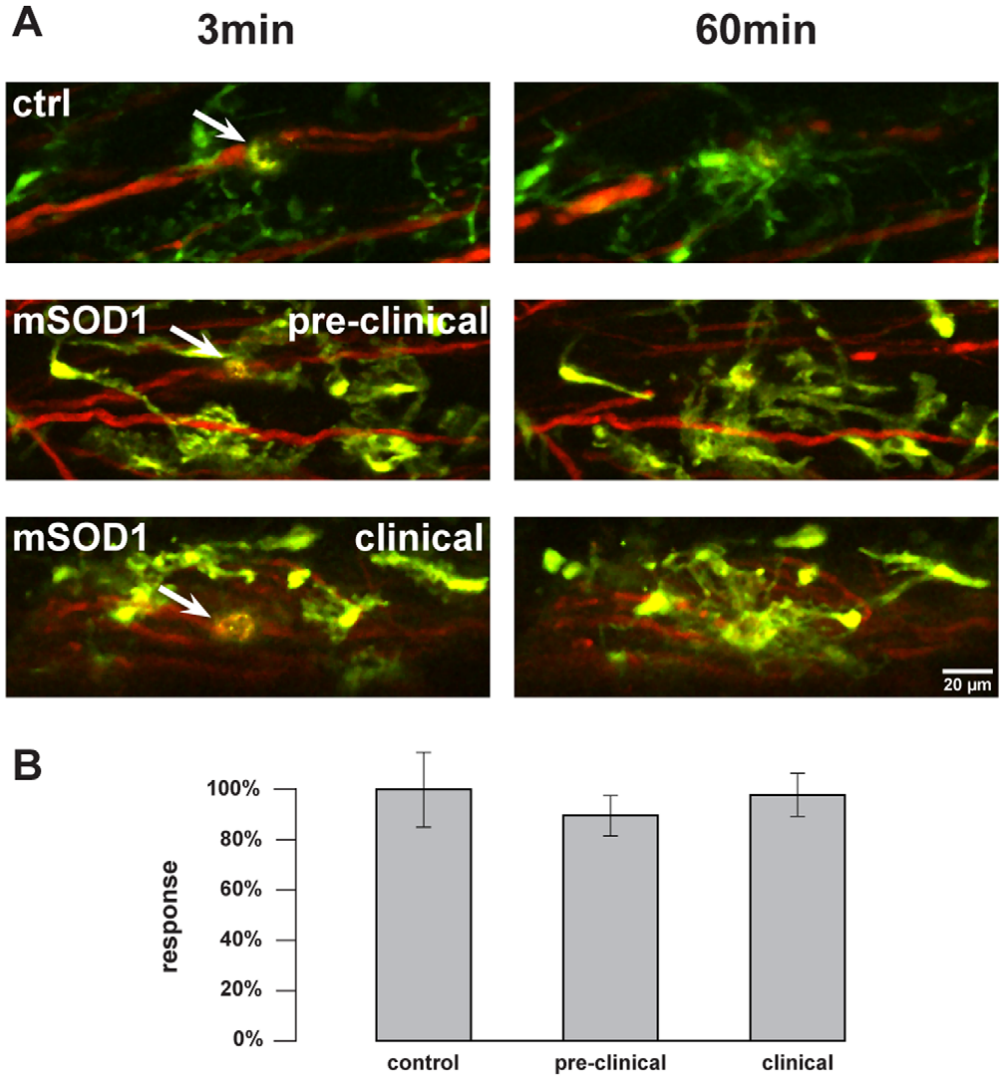
Microglial response in the dorsal column of the spinal cord during SOD1^G93A^ disease course. Microglial reaction towards laser-induced single axon transections within the spinal cord dorsal column (superficial layers as a sensory part, carrying primarily ascending fibers). ***A***, Left images were taken 3 min after axonal transection (autofluorescence and arrow) in control and mutant (preclinical 60-day-old and advanced clinical 120-day-old) mice. Respective images (right) were taken 60 min post lesion. See also Video S7, S8, S9. ***B***, Quantification of microglial response to the injured site. No significant differences were observed between control and mSOD1 mice. Control mice were of corresponding age (60 to 140 days of age). Values are presented as mean ± SEM; ANOVA followed by Tukey test.

To analyze microglial behavior at different disease stages in clinically non-affected spinal cord tissue, another set of experiments was performed within superficial layers of the dorsal columns. These anatomic structures carry primarily ascending fibers. Microglial response towards single axon transections within spinal cord dorsal columns was independent of the stage of the SOD1^G93A^ disease (Fig. 3; Video S7, S8, S9). Laser-induced lesions led to similar microglial reactions in mutant and control mice (preclinical: 89.72±8.13%, n = 5 mice; clinical: 97.83±8.71%, n = 6; compared to controls [20], set as 100±14%, n = 8). The comparison of control reactions in dorsal and lateral spinal cord revealed no significant differences of microglial reactivity within the two regions.

### Neurodegeneration and neuroinflammation in the peripheral nervous system of SOD1^G93A^

**Figure 4:**
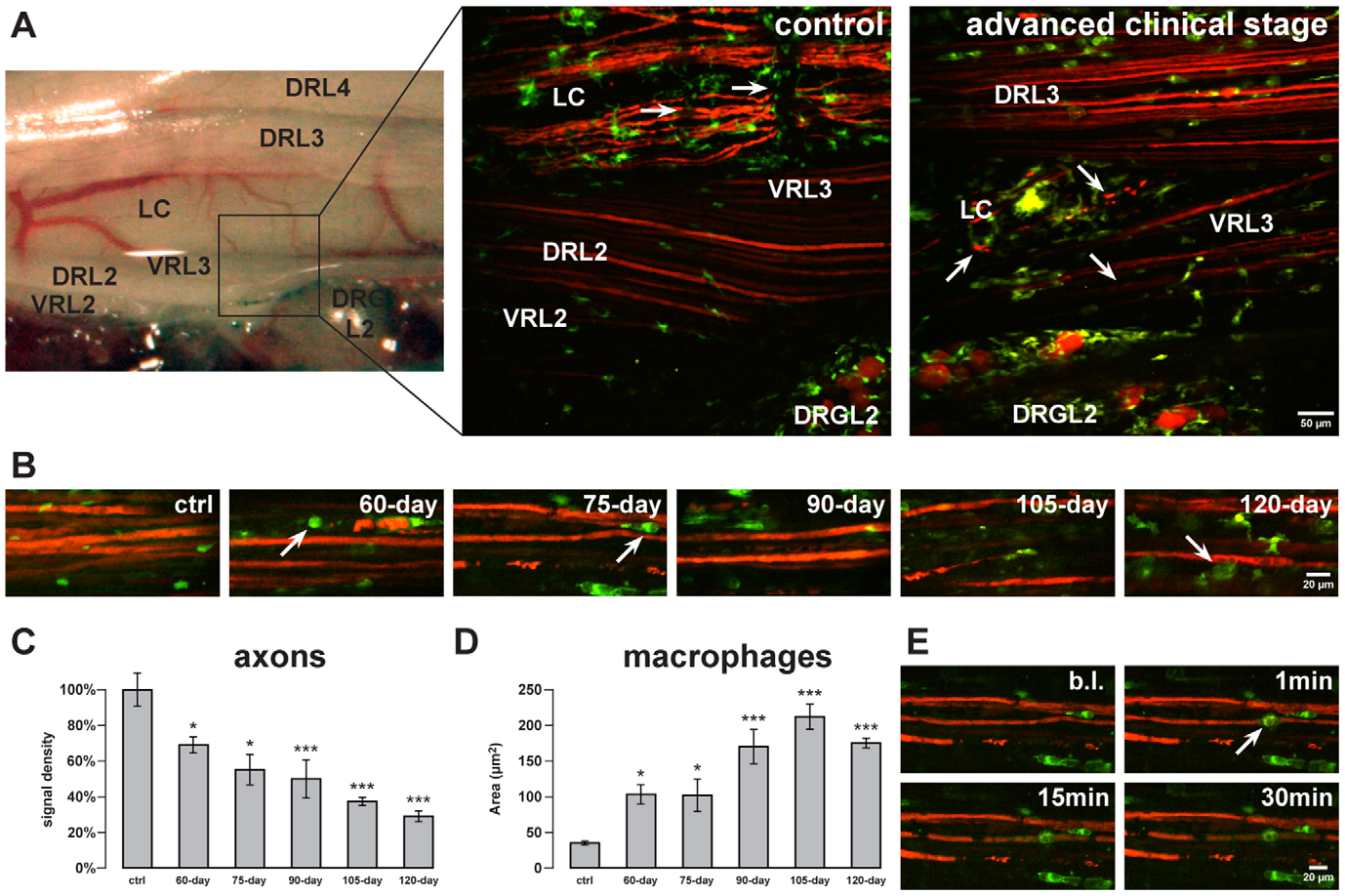
Degeneration of motor axons in spinal ventral roots during SOD1^G93A^ disease course. ***A***, Reflected light overview (left side) of the surgically exposed anatomic region. Central nervous system and proximal peripheral nervous tissue is depicted. A corresponding area, marked by the black box, was then recorded by 2P-LSM. The vessels in the lateral column (LC) of the spinal cord could also be recognized on the 2P-LSM image (marked by arrows). The image shows central axons and microglia of the lateral spinal cord as well as spinal root axons and spinal root macrophages. In an advanced clinical stage (120-day-old), axonal degeneration in the LC and in the recorded ventral root (VR), indicated by interruptions and swellings (some exemplary marked by arrows), were observed while dorsal root axons remain unaffected. ***B***, Increase of motor axon degeneration in the lumbar ventral roots from preclinical stages (60-day-old and 75-day-old), onset of disease (90-day-old) to clinical stages (105-day-old and 120-day-old). During this time course, macrophages changed their morphology forming foamy cytoplasm (some exemplary cells are marked by arrows). ***C***,***D***, Quantification of axonal signal density and cellular area of macrophages during the same disease stages that were shown in ***B***. Control mice were of corresponding age (60 to 140 days of age). ***E***, No morphological reaction of adjacent macrophages towards a laser-induced single axon transection (autofluorescence in green and arrow) was observed in the ventral root of preclinical (75-day-old) mice. The transection led to an acute axonal degeneration at both sides of the injury. Values are presented as mean ± SEM; ANOVA followed by Tukey test (*p<0.05, **p<0.01, ***p<0.001). LC, lateral column; DRL2-L4, dorsal root of the spinal segments L2-L4; VRL2-L3, ventral root of the spinal segments L2-L3; DRGL2, dorsal root ganglion of the spinal segment L2.

Early dysfunction of lower motor neurons in SOD1^G93A^ mice includes axonal deficits (e. g. accumulation of neurofilaments), slowing of axonal transport as well as retraction and loss of peripheral synapses at neuromuscular junctions (NMJs) long before first clinical symptoms appear (from 40-day-old animals onwards) [16]. This suggests an important and possibly even primary role of the distal peripheral nervous system (PNS) in promoting motor neuron degeneration, by e. g. retracting its nervous terminals [16], [17]. By exposing spinal roots and respective dorsal root ganglia, time-lapse 2P-LSM imaging was applied to analyze the role of macrophage-related neuroinflammation in peripheral axons. Pseudounipolar neurons in dorsal root ganglia, axons in spinal roots and surrounding macrophages were recorded simultaneously under above mentioned *in vivo* conditions. Fig. 4*A* depicts an overview of the exposed area of the central and peripheral nervous system in control and in an advanced clinical stage mouse (120-day-old). The latter contains degenerated central descending axons and degenerated lower motor axons in the spinal lateral column and ventral root, respectively.

During the course of the disease, lower motor axons in the lumbar ventral roots continuously degenerated (Fig. 4*B*). This was quantified by measuring signal density of EYFP-labeled axonal structures in comparable anatomic regions as mentioned previously. Even in preclinical stages (60-day-old and 75-day-old), signal density was significantly lower than in control mice suggesting an early axonal degeneration in the PNS. Signal density decreased with age from 60-day-old to 120-day-old animals (control set as 100±9.6%, n = 10 mice; 60-day-old: 69.3±4.9%, n = 9, p<0.05; 75-day-old: 55.6±8.8%, n = 5, p<0.05; 90-day-old: 50.5±11%, n = 6, p<0.001; 105-day-old: 37.9±2.6%, n = 6, p<0.001; 120-day-old: 29.7±3.4%, n = 9, p<0.001) (Fig. 4*C*).

During the same time course, macrophages changed their morphology forming foamy cytoplasm indicating an intracellular accumulation of abundant lipid: a sign of phagocytosis of degenerated myelinated tissue (Fig. 4*B*). Accordingly, the cellular volume of macrophages increased indicated by an increase of the cellular 2-dimensional area in maximum intensity projections (MIPs) (control: 37.02±2.78 µm^2^, n = 38 single cells in 10 mice; 60-day-old: 104.63±14.46 µm^2^, n = 41 cells in 9 mice, p<0.05; 75-day-old: 103.58±23.45 µm^2^, n = 20 cells in 5 mice, p<0.05; 90-day-old: 171.24±24.71 µm^2^, n = 26 cells in 6 mice, p<0.001; 105-day-old: 212.48±18.37 µm^2^, n = 36 cells in 6 mice, p<0.001; 120-day-old: 175.97±11.98 µm^2^, n = 65 cells in 9 mice, p<0.001) (Fig. 4*D*).

In contrast to microglia in the CNS, macrophages of control and SOD1^G93A^ mice did not react towards laser-induced single axon transections that led to acute symmetrical disintegration of ventral root axons (Fig. 4*E*). Also in peripheral nerves, macrophages did not react towards similar axon transections (data not shown). Simultaneous time-lapse recordings of adjacent areas of the central and peripheral nervous system, involved in the pathophysiology of mSOD1, show the difference in cellular activity between microglia and macrophages (clinical stage, Video S10).

**Figure 5:**
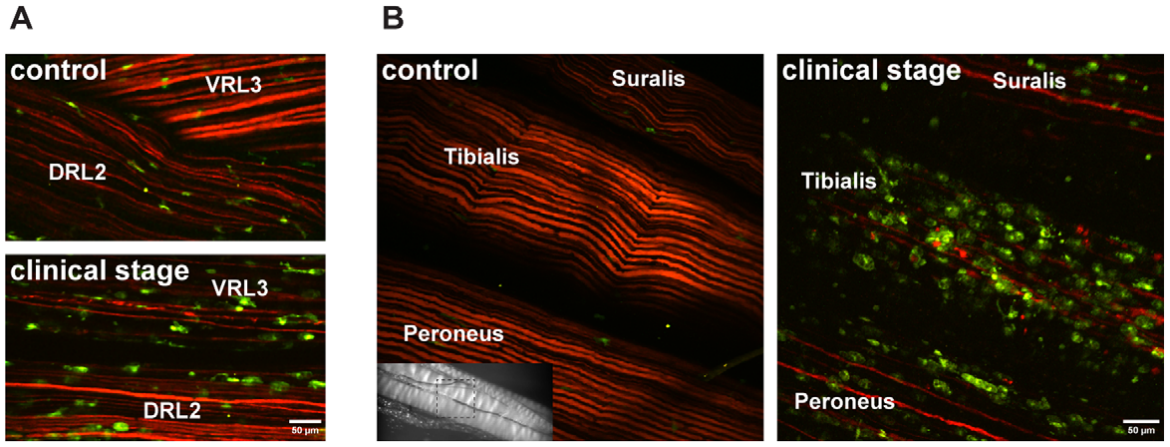
Degeneration of peripheral nerves during the disease course in SOD1^G93A^ mice. ***A***, Exemplary simultaneous 2P-LSM recordings of axons and macrophages in ventral and dorsal lumbar roots in wild-type and advanced clinical (120-day-old) mice. Degeneration of motor axons in the ventral root and increase of the cellular volume of macrophages in the ventral and dorsal roots were observed in the mutant mouse. ***B***, Distinct degeneration of peripheral axons in a 120-day-old SOD1^G93A^ mouse (clinical stage), shown by simultaneous recording of the 3 major nerves originating from the sciatic nerve. The tibial nerve was more affected than the common peroneal nerve or the sural nerve. Degenerated axons were abundantly surrounded by foamy macrophages. The imaged region is indicated in the insert (CCD camera image). Note that EYFP fluorescence is depicted with a red color table. DRL2, dorsal root of the spinal segment L2; VRL3, ventral root of the spinal segment L3.

Subtle involvement of sensory axons has been described in ALS [31], [32]. To test whether neurodegeneration also takes place in sensory roots of SOD1^G93A^ mice, simultaneous 2P-LSM recordings of ventral and dorsal lumbar roots were performed (Fig. 5*A*). Interestingly, foamy transformed macrophages could also be observed in dorsal roots of advanced clinical mice (120-day-old). However, direct proof of axonal degeneration, e. g. axonal interruptions, irregularities and swellings, was not found indicating only subtle degenerative changes (Fig. 5*A*).

**Figure 6:**
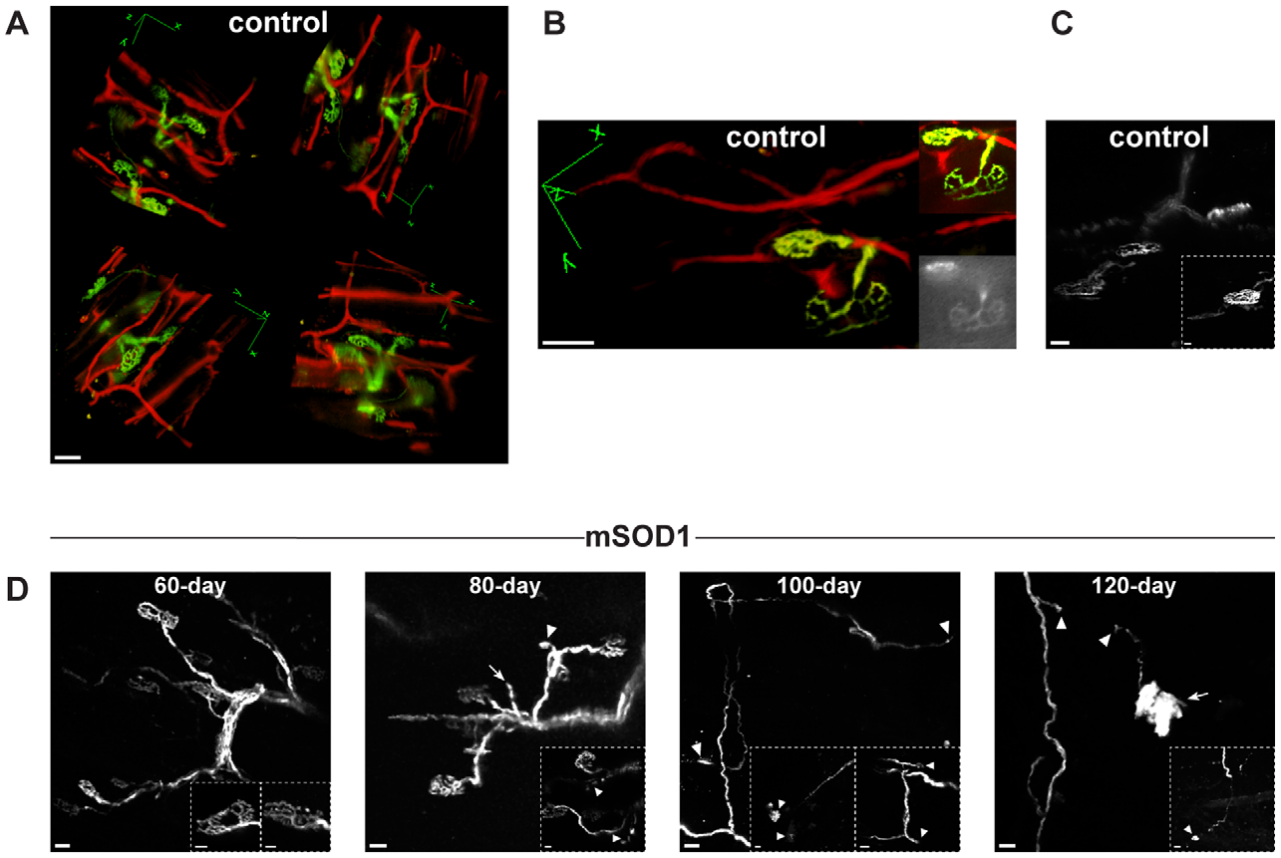
Degeneration of neuromuscular junctions (NMJs) during SOD1^G93A^ disease course in a muscle innervated by slow-motor neurons. Exemplary motor axons and NMJs of musculus tenuissimus (containing mainly slow twitch fibers) were imaged in control and SOD1^G93A^ (mSOD1) mice. EYFP fluorescence is depicted with a green color table. The vascular network, labeled by central venous application of the dextran Texas Red, is depicted with a red color table. ***A,B*** *,* Control NMJs and the adjacent vascular network are shown in 3 dimensions (in ***A*** the same region from 4 different perspectives is depicted). The inserts in ***B*** are a maximum intensity projection (MIP, upper insert) and an epifluorescence image (lower insert) of the two NMJs. ***C*** *,* Respective motor axons of a complex of NMJs in a control mouse. Two adjacent NMJs of different morphology are shown in the insert. ***D*** *,* Degeneration of NMJs and their respective motor axons during the course of SOD1^G93A^ disease in musculus tenuissimus. 60-day-old: morphologically unchanged NMJs when compared to controls; two intact synapses are shown in the inserts. 80-day-old: degenerated (arrowhead) or missing synapses (arrow) at the end of some motor axons; interrupted axon terminals (arrowheads) are shown in the insert. 100-day-old: advanced degeneration of the recorded axon complexes (including the inserts), indicated by missing synapses (arrowhead on the right side of the overview and arrowheads in the right insert), structures of broad-spectrum fluorescence (arrowhead on the left side of the overview and arrowheads in the left insert) and aberrant growth of axons. 120-day-old: interruption of a motor axon (arrowheads) and structures of broad-spectrum fluorescence (arrow, arrowhead in the insert). Note that musculus tenuissimus is innervated mainly by slow-motor neurons. These motor neurons show signs of NMJ denervation in SOD1^G93A^ mice at approximately onset of disease. Scale bars, 20 µm; inserts, 10 µm.

Retrograde axonal degeneration, initiated by abrupt distal pruning of selective motor neuron axons, is assumed to be critical in the development of motor neuron degeneration [16]. To study distal neurodegenerative processes in SOD1^G93A^ mice, we performed 2P-LSM recordings of more distal PNS regions (Fig. 5*B* and 6). Simultaneous recordings of the 3 major nerves originating from the sciatic nerve revealed different axonal vulnerabilities (Fig. 5*B*). Depending on the efferent fraction, the tibial nerve was more affected than the common peroneal nerve or the sural nerve in clinical stages of the disease. Similar to the neurodegeneration in ventral roots, degenerated axons were surrounded by a high number of foamy macrophages, consistent with previous data [33]. This observation again indicates phagocytosis of myelinated tissue. In control nerves, only a few macrophages, relatively small in size, were observed (Fig. 5*B*).

Early axonal vulnerability in motor neuron diseases is linked to axonal transport impairment [16], [34], [35]. This “dying back” mechanism is associated with a selective vulnerability of axonal transport leading to synaptic vesicle stalling and subsequent synapse loss [16]. Fast-fatiguable (FF) motor axons are affected very early (first detectable in nearly 40-day-old SOD1^G93A^ mice), whereas weakening, followed by loss of fast fatigue-resistant (FR) axons and weakening of slow (S) axons coincide with the onset of the clinical phase and progression during advanced clinical stages of the disease, respectively [16]. To determine clinically associated degeneration of motor axons and their respective NMJs, musculus tenuissimus consisting of slow twitch fibers, mainly innervated by FR and S axons, was recorded *in vivo*. This muscle, provided with retaining functions, is located proximal to the biceps femoris muscle (therefore also known as the short head of the biceps) [36]. Fig. 6*A and B* show control NMJs and the adjacent vascular network, labeled by central venous application of the dextran conjugate Texas Red, within the muscle in 3 dimensions. Some synapses were surrounded by thin vessels (an example is shown in Fig. 6*B*). Respective motor axons of a complex of NMJs are shown in Fig. 6*C*. During the course of the disease, the NMJs and their respective motor axons progressively degenerated (Fig. 6*D*). NMJs began to degenerate shortly before the onset of the disease (80-day-old) indicated by missing synapses at motor axon terminals and by interrupted axon terminals. Around onset of disease, a complete degeneration of some NMJ complexes was observed (Fig. 6*D*). In addition, structures of broad-spectrum autofluorescence replaced degenerated synapses. In addition, aberrant growth of axonal structures could be observed suggesting compensatory attempts of motor axons to reinnervate muscle. Distinct accumulations of similar autofluorescence were also observed in advanced clinical stages (Fig. 6*D*). In these stages, already numerous NMJs were degenerated.

## Discussion

Microglia substantially contribute to motor neuron death and disease progression in mSOD1 mouse models for ALS [4], [5], [6], [11], [14], [15]. Our study using the SOD1^G93A^-model provides substantial *in vivo* evidence that microglia-mediated inflammation in the CNS comprises two different phases with fluent transition: i) in preclinical stages, microglia is highly reactive as indicated by an enhanced injury-induced response including rapid soma migration and early phagocytic activity; ii) in clinical stages, activated ameboid microglia lose their target-directed response, but instead show spontaneous activity as a gain of function and a loss of its role as highly dynamic surveillants of the CNS [19]. The increase of microglia presence, the ameboid transformation, the loss of tissue surveillance and the decrease of injury-directed process movements, all of them mainly occurring during the clinical course, emphasize the contribution of microglia to disease progression in mSOD1 animals in the late phase of disease. This is consistent with previous data [4], [11].

The enhanced microglial reactivity towards focal lesions in preclinical stages, however, supports the role of microglia as a ‘double-edged sword’. Erroneous (overactive) response to tissue injuries may represent early harmful microglial activity that promotes axonal and neuronal degeneration. On the other hand, rapid response to local damage leading to early clearing from debris and neurotoxic molecules may also be beneficial and may even attenuate ongoing focal neurodegeneration. Previous findings by selective gene silencing [4] support the second interpretation of our observations.

Several factors may contribute to the compromised function of mSOD1-microglia in clinical stages such as cytoskeletal transformation towards an activated phenotype, accompanied by alterations of transcriptional and non-transcriptional factors and their functional consequences [23], [37]. These alterations may particularly include changes of the expression profile of receptors and enzymes which bind signal molecules, involved in sensing tissue abnormalities by microglia, such as ATP [18], [21], [38] and Nitric oxide (NO) [20], [39]. A purinergic contribution to and an important role of NO in microglia-mediated motor neuron degeneration has already been demonstrated [40], [41], [42], [43]. In particular, increase of purinergic receptors on microglia [40] and enhanced inducible NO-synthase activity in microglia of the spinal cord [42], [43] are correlated with an increased activity of microglia. Interestingly, earlier studies provided evidence that microglial response to local injuries depends on ATP and NO gradients rather than on overall ATP or NO concentration [18], [20], [21], [38]. In the context of chronic neurodegenerative diseases, e. g. ALS, constantly increased ATP/NO tissue levels as a consequence of ongoing tissue destruction would impede the rapid development of local ATP/NO gradients. This may explain, on a molecular level, the reduced microglial response to axonal injuries in clinical stages. In addition, constantly high tissue levels of signal molecules may lead to a decreased expression of respective receptors [38], which by themselves would down-regulate the response of microglia.

Even though the reported abnormalities in microglial behavior could be due to an abnormally high level of mutated SOD1 *per se* in the SOD1^G93A^ mouse, previous data analyzing mice which overexpress the human wild-type SOD1 demonstrate that at least the up-regulation of pro-inflammatory cytokines in microglia is specifically due to the presence of the mutated gene [44]. Whether the observed morphological and behavioral changes of microglia is due to the mSOD1-protein overexpression within microglia cells or a response to local environmental changes during the disease course, or both is a longstanding matter of debate. Our data analyzing SOD1^G93A^ microglia reaction in the non-affected part of the spinal dorsal columns carrying primarily ascending fibers, strongly suggest that environmental changes are at least a prerequisite for microglia-mediated neuroinflammation. The unaltered microglial morphology and reaction to axonal injury throughout the course of the disease in a sensory part of the spinal cord emphasizes a secondary role of microglia-mediated neuroinflammation in the pathophysiology of mSOD1. Furthermore, the finding that mutant microglia show no aberrant inflammatory behavior in non-affected spinal tissue supports the non-cell-autonomous pathophysiology of mSOD1.

The process of microglial transformation is accompanied by changes of the cellular expression profile. This leads to an increase of cytokine and radical release [23] which, by itself, promotes further neuroinflammatory activity also in other cell types, e. g. astrocytes. In this context, even extracellularly located mSOD1 proteins sustain or trigger the ongoing neuroinflammation [45], [46]. However, our data do not distinguish between an aberrant behavior of microglia due to the mutant SOD1 expression or to an activated state of microglia as response to chronic environmental changes.

Furthermore, we observed spontaneous formation of cell clusters in mSOD1 mice. This has not been described in WT or control animals in a number of recent studies including *in vivo* imaging approaches [18], [19], [20], [21], [23]. This observation indicates that a crucial role of microglia which is permanent tissue surveillance, is impaired in mSOD1 animals. In support of this, a recent study shows formation of multinucleated giant cells through fusion of microglial cells in the spinal cord of clinically affected mSOD1 mice [47]. On a cellular level microglia undergo, at least in part, structural abnormalities during the course of mSOD1 disease which are indicative of cellular degeneration [47].

Recent data show that only a low number of CST fibers are found in the lateral white matter (lateral CST fibers are mostly found in the dorsal portion of the lateral column) [48]. In addition, the diameter of CST fibers is relatively small (even if a population of larger fibers was found in the lateral CST, more than 90% of the CST fibers were less than 1.5 µm in diameter) [48]. Therefore, the high rate of the observed degeneration of apparently relatively large axons in lateral spinal cord suggests ongoing neurodegeneration within this part of the spinal cord white matter that includes more fiber tracts than the pyramidal tracts. Whether additional degeneration of the extrapyramidal system plays a role in the pathophysiology of mSOD1 needs further investigation. Interestingly, extrapyramidal involvement seems to play a role in stiffness and balance impairment in a subset of ALS patients [49].

Different to microglia-mediated neuroinflammation in the CNS, macrophages do not respond to focal injuries in the PNS. Early involvement of macrophages includes transformation to ‘foamy’ macrophages which indicates a passive role of clearing debris, particularly lipid-rich myelin. This is in line with previous findings showing changes in the expression profile of myelin-laden macrophages towards a dominance of anti-inflammatory proteins [50]. Nonetheless, the functional consequence of the apparently contradictory up-regulation of the activity marker CD11b in peripheral macrophages during mSOD1 disease course, recently shown by immunohistochemical analysis [51], has to be elucidated. An active role of macrophages in chronic axonal neurodegeneration by, for instance, the release of toxic radicals or cytokines can not be excluded and remains to be clarified. However, other data, obtained by characterization of macrophage activation during disease progression, also suggest phagocytic removal of debris as the primary role of macrophages in the PNS emphasizing their non-detrimental function [33]. These findings are indications of a primarily neurodegenerative process in the PNS without substantial inflammatory contributions. They even suggest a rather pro-regenerative role of activated macrophages. The data strengthen the idea of distinct inflammatory activities in the CNS versus PNS, one with a dominant neuroinflammatory response in the CNS and another with primarily degenerative processes in the PNS.

Several studies suggested that axonal degeneration in the PNS including early loss of NMJs is a primary pathophysiological event in fALS mice [16], [17], [52], [53], [54]. Immunohistochemical investigations have revealed that axonal loss in lumbar ventral roots in preclinical stages occurs earlier than cell body loss in the ventral horn of the spinal cord [55]. Dysfunction of distal parts of the motor neuron apparently precedes the onset of disease. Our data analyzing axonal degeneration in the PNS supports this hypothesis by showing axonal loss in ventral roots of the lumbar spine as early as from day 60 onwards. However, neither can we, based on the presented *in vivo* data, strictly confirm a ‘dying back’ mechanism nor a ‘distal axonopathy’. Loss of neuromuscular junctions appears strongly dependent on the type of muscle [16], [52]. In muscles mainly innervated by fast-fatigue-resistant and slow-motor neurons, such as the musculus tenuissimus, pruning of motor axons and the respective NMJs begins at symptom onset. In muscles mainly innervated by fast-fatigable motor neurons, loss of NMJs is already observed in presymptomatic stages.

Selective motor neuron degeneration arises from the convergence of a series of pathophysiological factors [5], [56], [57], [58]. Neuroinflammation apparently goes along with neurodegenerative processes. Using an *in vivo* imaging approach that allows us to visualize cell-cell interaction over a time course of hours, we present evidence for distinct mSOD1-induced inflammatory activities in the CNS versus the PNS. We suggest that microglia-mediated neuroinflammation in the CNS promotes neurodegeneration in clinical stages of the disease while cellular changes of macrophages in the PNS support a passive role. Neurodegeneration with axonal degeneration and loss of NMJs, considered a primary event in ALS pathogenesis, may underlay the hitherto limited response of animal models or human trials to anti-inflammatory therapies.

## Supporting Information

Video S1 **Microglial reaction towards single axon transection in the spinal cord lateral column of control mice.** The experiments were performed in double transgenic mice expressing EGFP in microglia and EYFP in projection neurons. Microglia are depicted in green while, for better visualization and distinction, axons are shown in red. All videos are arranged such that rostral is to the left side. This video sequence (105 minutes length) lasts from 45 minutes before to 60 minutes after a laser-induced axonal transection (the injury was set at 0 min). It shows microglial reaction towards the site of the injury (note the autofluorescence as speckles in the image center; marked by arrow). Surrounding microglia immediately extended their processes forming a shielding ring around the injury. The transection led to an acute axonal degeneration at both sides of the injury, indicated by interruptions and bulb-like swellings. Within the recorded time-lapse sequence of 60 minutes after injury, reacting microglial cells polarized and began to migrate in an ameboid manner towards the injured axon. No phagocytic activity of microglia was observed within the first hour after the injury. Note the monocytes within the blood vessels. The control mouse was 90 days of age. The video sequence corresponds to a time-lapse series of 53 stacks of images. Each of these stacks is shown as a maximum intensity projection (MIP). The frame rate of the video is about 2 minutes per MIP. Scale bar, 20 µm.
(MOV)

Video S2 **Detailed microglial reaction towards single axon transection in the spinal cord lateral column of control mice.** This video sequence (75-minute) lasts from 15 minutes before to 60 minutes after a laser-induced axonal transection. It shows microglial reaction towards the injured site (note the autofluorescence; arrow) and the acute axonal degeneration in more detail when compared with video S1. The control mouse was 120 days of age. The frame rate of the video is 65 seconds per MIP. Scale bar, 20 µm.
(MOV)

Video S3 **Enhanced response and early migration of microglia towards a single axon transection in the lateral column of a preclinical SOD1^G93A^ mouse.** This video sequence (195-minute) lasts from 15 minutes before to 180 minutes after a laser-induced axonal transection in a 60-day-old mutant mouse. It shows an increased response of microglia towards the injured site (note the autofluorescence; arrow). The response comprised a rapid movement of cell soma starting within a few minutes after the injury. An enhanced and early (starting within the first hour after the injury) phagocytic activity of microglia, indicated by numerous engulfments within and around the injured site, could also be observed. The frame rate of the video is about 90 seconds per MIP. Scale bar, 20 µm.
(MOV)

Video S4 **Early phagocytosis after a single axon transection in the lateral column of a preclinical SOD1^G93A^ mouse.** This video sequence (90-minute) lasts from 15 minutes before to 75 minutes after an axonal transection in a 75-day-old mutant mouse. It shows, similar to the Video S3, an increased response of microglia towards the injured site (note the autofluorescence in the image center; arrow). The response comprised an early phagocytic activity of microglia at both sides of the injury. Note the engulfment and the subsequent phagocytosis of an EYFP-containing bulb-like swelling of the transected degenerating axon about 30 µm to the right to the injured site (arrow). The frame rate of the video is 85 seconds per MIP. Scale bar, 20 µm.
(MOV)

Video S5 **Lack of microglial reaction after single axon transection in the lateral column of a clinical SOD1^G93A^ mouse.** This video sequence (145-minute) lasts from 25 minutes before to 120 minutes after an axonal transection in a 120-day-old mutant mouse. It shows a highly reduced microglial reaction towards the tissue lesion (note the autofluorescence in the image center; arrow). The axonal transection led to degeneration of the axon at both sides of the injury without any apparent contribution of microglia. Note the increased microglial activity including numerous engulfments and continuous ameboid migration independent of the injury setting. The frame rate of the video is 115 seconds per MIP. Scale bar, 20 µm.
(MOV)

Video S6 **Spontaneous activity of microglia in the lateral column of a clinical SOD1^G93A^ mouse.** This video sequence (60-minute) shows enhanced spontaneous activity of ameboid microglia in a 120-day-old mutant mouse. This activity included permanent ameboid migration (arrows) and permanent phagocytic processes. The frame rate of the video is 128 seconds per MIP. Scale bar, 20 µm.
(MOV)

Video S7 **Microglial reaction towards a single axon transection in the spinal dorsal column of a control mouse.** This video sequence (80-minute) lasts from 7 minutes before to 73 minutes after an axonal transection (set at 0 min). It shows microglial reaction towards the site of the injury (note the autofluorescence; arrow). Surrounding microglia immediately extended their processes forming a shielding ring around the injury. The transection led to an acute axonal degeneration at both sides of the injury. Note the monocytes within the blood vessels. The control mouse was 90 days of age. The frame rate of the video is 80 seconds per MIP. Scale bar, 20 µm.
(MOV)

Video S8 **Microglial reaction towards a single axon transection in the spinal cord dorsal column of a preclinical SOD1^G93A^ mouse.** This video sequence (80-minute) lasts from 20 minutes before to 60 minutes after an axonal transection in a 60-day-old mutant mouse. It shows, similar to the control mouse (Video S7), microglial reaction towards the site of the injury (note the autofluorescence; arrow). The frame rate of the video is about 1 minute per MIP. Scale bar, 20 µm.
(MOV)

Video S9 **Microglial reaction towards a single axon transection in the spinal cord dorsal column of a clinical SOD1^G93A^ mouse.** This video sequence (64-minute) lasts from 4 minutes before to 60 minutes after an axonal transection in a 120-day-old mutant mouse. It shows, similar to the Video S7 and S8, microglial reactions towards the site of the injury (note the autofluorescence; arrow). Note the monocytes within the blood vessel on the left side. The frame rate of the video is 116 seconds per MIP. Scale bar, 20 µm.
(MOV)

Video S10 **Simultaneous recording of microglia and macrophages in a clinical SOD1^G93A^ mouse.** This video sequence (45-minute) shows spontaneous microglial activity and cluster formation in the lateral column of the spinal cord (upper part) compared to minimal morphological movements of macrophages in an adjacent ventral root (lower part) in a 105-day-old mutant mouse. The frame rate of the video is about 73 seconds per MIP. Scale bar, 20 µm.
(MOV)
